# Micronutrient powders to combat anaemia in young children: do they work?

**DOI:** 10.1186/s12916-017-0998-y

**Published:** 2018-01-22

**Authors:** Hans Verhoef, Emily Teshome, Andrew M. Prentice

**Affiliations:** 1 MRC Unit The Gambia, Atlantic Boulevard, Fajara, Gambia; 2 MRC International Nutrition Group, London School of Hygiene and Tropical Medicine, Keppel Street, London, WC1E 7HT UK; 3 Division of Human Nutrition and Cell Biology and Immunology Group, Wageningen University, P.O. Box 338, 6700AH Wageningen, The Netherlands

**Keywords:** Iron, Anaemia, Iron deficiency, Fe(III)-EDTA, Food, Fortified, Meta-analysis, Child, Preschool

## Abstract

In 2016, the World Health Organization (WHO) recommended point-of-use fortification of complementary foods with iron-containing micronutrient powders to improve iron status and reduce anaemia in children at risk of anaemia. This recommendation continues to be debated. In a recent trial among Kenyan children aged 12–36 months, we found no evidence that daily point-of-use fortification was efficacious in improving haemoglobin concentration or plasma iron markers. An updated meta-analysis indicated that, on average, in an arbitrarily selected setting and with adherence as obtained under trial conditions, one may expect a small increase in haemoglobin concentration in preschool children, with the upper limit of the 95% CI virtually excluding an effect beyond 5.5 g/L. In the present paper, we elaborate on the interpretation of these findings and the meta-analyses that formed the basis for the WHO guidelines. In particular, we draw attention to the phenomenon that small group differences in the distribution of continuous outcomes (haemoglobin concentration, ferritin concentrations) can give a false impression of relatively large effects on the prevalence of the dichotomised outcomes (anaemia, iron deficiency).

Please see related articles: https://bmcmedicine.biomedcentral.com/articles/10.1186/s12916-017-0839-z, https://bmcmedicine.biomedcentral.com/articles/10.1186/s12916-017-0867-8

## Background

Point-of-use fortification of foods is recommended by the World Health Organization (WHO) [1] as an alternative to mitigate or overcome the constraints associated with supplementation and industrial food fortification. In this new approach, powders containing a mixture of vitamins and minerals are supplied as small, single-serving packets, the contents of which can be mixed into semi-solid food prior to consumption.

In a recent placebo-controlled trial among Kenyan children aged 12–36 months, we found no evidence that daily point-of-use fortification with either 3 mg iron as NaFeEDTA or 12.5 mg iron as ferrous fumarate was efficacious in improving haemoglobin concentration or plasma iron markers [2]. As discussed in a commentary [3], the decline in anaemia prevalence over time that we observed in all intervention groups may have been due to premedication with anti-malarial and anti-helminth medication at baseline, or due to the vitamin A or zinc content in the fortification powders. However, attribution of such time trends is impossible since the observed effects may also have been due to various other factors, including seasonal variation in nutrient status or infections, age changes or regression to the mean. Thus, these temporal changes in anaemia prevalence cannot be per se interpreted as evidence to support the use of micronutrient powders in an effective intervention strategy. Reviews of the impact of multi-micronutrient powders on indicators other than iron deficiency or anaemia have been inconclusive [4, 5], and unfortunately there is little evidence from randomised trials to show that point-of-use fortification leads to improved vitamin A or zinc status [6].

## Summary measures of intervention effect

The effect of an intervention for any person is best defined as the difference between the outcomes as a result of receiving the intervention and those that would have resulted had the intervention been denied [7]. Under such a counterfactual view of causality, an effect can be operationally estimated as the difference in endpoints between groups concurrently randomised to verum (i.e. to the active substance under investigation) or control. In our trial, compared to placebo, point-of-care fortification with NaFeEDTA at the end of intervention led to a reduction in the prevalence of iron deficiency by 20.1% (from 44.6% to 24.5%). However, effects on iron deficiency should be interpreted with caution since a small group difference in the distribution of a continuous outcome can misleadingly result in a relatively large difference in prevalence of the dichotomised outcome, whether expressed as a relative or absolute difference. Thus, point-of-use fortification with NaFeEDTA improved geometric mean plasma ferritin concentrations by only 4 μg/L (from 29.7 to 33.7 μg/L), which may seem much less impressive than suggested by the observed 20.1% decrease in prevalence of iron deficiency, an outcome variable that was derived by dichotomising plasma ferritin concentration.

Similar issues arise in the interpretation of effect estimates of the meta-analyses that formed the basis of the WHO guidelines, which showed absolute effects on haemoglobin and ferritin concentrations that may seem much less impressive than the corresponding relative reductions in anaemia and iron deficiency [1]. For example, in the WHO meta-analysis, in children aged 2–12 years, point-of-care fortification with multiple micronutrient powders increased haemoglobin concentration by 3.4 g/L (95% CI 0.9–5.8 g/L) as compared to a reduction in anaemia risk by 34% (95% CI 12–51%) [1]. In trials and meta-analyses, priority should be given to effects on continuous outcomes. Relative effects on dichotomised outcomes can be useful to allow extrapolation of trial results over time and in different settings, but they should be given secondary importance, and extrapolated results should be re-converted to the continuous outcome to appreciate the true public health gains.

## Future directions and conclusions

Our meta-analysis suggested a small gain in haemoglobin concentration in most trials, indicating that point-of-use fortification with iron-containing micronutrient powders provides some benefit across different settings [2]. On average, in an arbitrarily selected setting, and with an adherence as obtained under trial conditions, one may expect an increase in haemoglobin concentration by only 3.9 g/L, with the upper limit of the 95% CI virtually excluding an effect beyond 5.5 g/L; the attenuated effect that is likely to be achieved under real-world conditions is even lower.

The recently published Global Burden of Disease estimates [8] have confirmed that iron deficiency anaemia is by far the most prevalent micronutrient issue worldwide and remains a scourge of humanity. The motive behind our robust interpretation of the low efficacy of multiple micronutrient powders is to encourage researchers, research funders and policymakers to continue to investigate the underlying reasons for the low efficacy. For instance, we have strong evidence that hepcidin blocks iron absorption in young children in response to even very low levels of inflammation (Prentice et al., submitted). Alleviation of such inflammation, regardless of the means, would likely improve the efficacy of all methods used to increase the iron content in children’s diets.

## References

[1] World Health Organization (2016). WHO Guideline: Use of Multiple Micronutrient Powders for Point-of-Use Fortification of Foods Consumed by Infants and Young Children Aged 6–23 Months and Children Aged 2–12 Years.

[2] Teshome EM, Andang’o PEA, Osoti V, Terwel SR, Otieno W, Demir AY, Prentice AM, Verhoef H (2017). Daily home fortification with iron as ferrous fumarate versus NaFeEDTA: a randomised, placebo-controlled, noninferiority trial in Kenyan children. BMC Med.

[3] Wieringa FT (2017). Micronutrient powders to combat anemia in young children: does it work?. BMC Med.

[4] De Pee S, Irizarry L, Kraemer K, Jefferds MED. Micronutrient powder interventions: the basis for current programming guidance and needs for additional knowledge and experience. In: Home Fortification with Micronutrient Powders. Sight and Life: Home Fortification Technical Advisory Group; 2013. p. 51–6. http://www.hftag.org/assets/downloads/hftag/Sight%20&%20Life%20supplement%20distributed%20at%20ICN%202013_FULL.pdf. Accessed 24 Oct 2017

[5] De-Regil LM, Suchdev PS, Vist GE, Walleser S, Peña-Rosas JP (2011). Home fortification of foods with multiple micronutrient powders for health and nutrition in children under two years of age. Cochrane Database Syst Rev.

[6] Silva LLS, Augusto RA, Tietzmann DC, Sequeira LAS, Hadler MCCM, Muniz PT, de Lira PIC, Cardoso MA, ENFAC Working Group (2017). The impact of home fortification with multiple micronutrient powder on vitamin A status in young children: a multicenter pragmatic controlled trial in Brazil. Matern Child Nutr.

[7] Senn S (2008). Statistical Issues in Drug Development.

[8] GBD 2016 Disease and Injury Incidence and Prevalence Collaborators (2017). Global, regional, and national incidence, prevalence, and years lived with disability for 328 diseases and injuries for 195 countries, 1990–2016: a systematic analysis for the Global Burden of Disease Study 2016. Lancet.

